# Intelligent Traffic Monitoring through Heterogeneous and Autonomous Networks Dedicated to Traffic Automation

**DOI:** 10.3390/s22207861

**Published:** 2022-10-16

**Authors:** Eduard Zadobrischi

**Affiliations:** 1 Department of Computers, Electronics and Automation, Faculty of Electrical Engineering and Computer Science, “Stefan cel Mare” University, 720229 Suceava, Romania; eduard.zadobrischi@usm.ro; 2 Department of Computer Science, Technical University of Cluj-Napoca, 400027 Cluj-Napoca, Romania

**Keywords:** traffic modelling, vehicle classification, intelligent system transportation model, traffic congestion, critical transportation infrastructure, transportation safety

## Abstract

In direct line with the evolution of technology, but also with the density of vehicles that create congestion and often road accidents, traffic monitoring systems are parts that integrate intelligent transport systems (ITS). This is one of the most critical elements within transport infrastructures, an aspect that involves extremely important financial investments in order to collect and analyze traffic data with the aim of designing systems capable of properly managing traffic. Technological progress in the field of wireless communications is advancing, highlighting new traffic monitoring solutions, and the need for major classification, but proposing a real-time analysis model to guide the new systems is a challenge addressed in this manuscript. The involvement of classifiers and computerized detection applied to traffic monitoring cameras can outline extremely vital systems for the future of logistic transport. Analyzing and debating vehicle classification systems, examining problems and challenges, as well as designing a software project capable of being the basis of new developments in the field of ITS systems are the aim of this study. The outline of a method based on intelligent algorithms and improved YOLOv3 can have a major impact on the effort to reduce the negative impact created by chaotic traffic and the outline of safety protocols in the field of transport. The reduction of waiting times and decongestion by up to 80% is a valid aspect, which we can deduce from the study carried out.

## 1. Introduction

The expansion, in terms of the high number of vehicles, increases the degree of saturation and exceeds the capacity of the existing transport networks, reaching a severe congestion factor in several countries of the world [1]. In addition to this aspect, over 1.3 million victims are killed and another 50 million are injured annually due to road accidents, a pressing problem in our society. These aspects involve extremely high costs in terms of the country’s economy, with estimates showing that between 1 and 3% of the domestic product is affected [2]. However, building new additional infrastructure is a solution in most cases, but high costs and limited building space are defining factors of the current situation. It can be said that delays increase prohibitively when it comes to providing safety to service providers in the field of highway construction, additional facilities in order to maintain a high standard and decongest traffic throughout the duration of the works, and also to divert traffic to alternative routes. Approximately 90% of road accidents are caused by the error of human factors, through lack of attention, fatigue, delayed reactions, and lack of distributive attention [3]. Thus, a traffic monitoring system becomes an effective alternative in the process of warning of traffic congestion. Being an integral component within intelligent transport systems (ITS), it is used in the collection of traffic data, such as the number of vehicles, their types, or the speed of travel. Based on this collected information, traffic analyzers are carried out, later they are transmitted to the integrated systems at the road infrastructure level in order to predict future transport and improve transport [4]. In terms of safety and traffic monitoring applications, we have to consider the need for low latencies, reaching up to 20 ms, in order to detect collisions and pre-crash.

These aspects have highlighted that the delivery of data packets and communication according to standards varies up to approximately 300 m [5]. Even if national transport agencies are actively involved and invest huge amounts of money in order to develop, implement, and maintain traffic-monitoring systems [6,7], it is not enough, and new alternatives are needed. It can be said that one of the main functionalities of a traffic monitoring system is vehicle classification. This aspect is essential in view of the extremely significant technical challenges of investigating problems of a mechanical nature or cataloging certain vehicles as dangerous due to their age. The research carried out on the formation and spread of congestion or accidents also led to a first response, whereby vehicles with serious technical problems cause the loss of human lives and traffic congestion through the defects they have. Another important finding is that more than 93% of deaths caused globally occur in low- and middle-income countries that do not automatically have adequate road infrastructure and are unable to provide the best conditions for road users, although these countries contribute only about 60% of the total number of vehicles in the world [8]. In other words, the precise classification of vehicles into several types is an extremely crucial aspect in order to operate the traffic efficiently, but also to plan it according to a predetermined algorithm according to the road sector. For example, information on the number of oversized vehicles on a highway section can be used to estimate the capacity of that section, and to plan part of the roadway maintenance processes. An important aspect dictated by the types of vehicles that use the running surface is the appearance and the geometric design that it must respect. Current developments and progress achieved through the implementation of automatic detection or learning technologies, as well as wireless communications, have given birth to new types of innovative systems in terms of vehicle classification and analysis. 

These new systems approaches, which classify vehicles with much greater precision, use advanced sensor units and hardware components to operate over extremely long distances. Within the manuscript, an exact analysis of the stage reached by the vehicle classification techniques and ways of approaching the problem is carried out, as well as the realization of some simulation scenarios that highlight an emerging solution on which the existing ITS systems could be based. The method of classification, the problems arising in the studies carried out, and the development of a new approach that increases the accuracy of classification will be systematically analyzed. The analyses and classifications will be carried out on three categories of approaches, those of the in-road-based, over-road-based, and side-road-based types, although in these situations, the systems developed up to this moment have reached the stage of revolutionary technologies and implemented direct communication between the road units and the infrastructure. The implementation of ITS systems that are based on intelligent algorithms for monitoring and streamlining traffic is another approach within the manuscript. It can be said that many intelligent systems based on V2X or 5G communications are installed in the testing phase in several areas of the world, including those responsible for analyzing some standard traffic monitoring and congestion loop detection networks [9], observing an appetite for vision-based vehicle classification techniques [10,11], and neglecting urgent classification solutions. The main purpose of the article is to highlight the need and usefulness of vehicle classification solutions in order to implement, at the infrastructure level, some devices that address and remedy current problems. Neglecting the aspects that lead to road accidents or traffic congestion leads to a precedent in terms of loss of human life. We consider relevant the study of the classification of vehicles, and depending on their category, their dimensions, or the speeds they reach, they should be directed differently according to certain classifiers. A comprehensive study of the approaches from the last decade is analyzed, and to lead to a result, a traffic monitoring solution via RF and Wi-Fi installed at the road infrastructure level will be proposed. 

We must not neglect the main aspect, that most of the current road infrastructures benefit from cameras installed at the level of traffic lights. This aspect can accurately analyze and measure the density of vehicles at each time interval, day and night. This can also help with designing simulation methods to validate models that analyze the similarities of the simulated traffic compared to the real one. All these aspects were not carried out in the same study; therefore, we consider the proposed approach through which classification methods are proposed, and this is carried out on the basis of captures made in real-time directly from video sensors mounted both in high-density urban areas, but also at the entrance to the suburbs of the cities, constitutes another point of view in this direction. The involvement of road infrastructure within an ITS system based on video processing and emerging communications between infrastructure and vehicles outlines a favorable framework for the development of complex autonomous systems. The simulation in critical conditions and environments with real training data that highlights an extremely exhaustive manner nevertheless creates a process through which future research in the direction of autonomous driving can be carried out. The component that differentiates the current approach from others consists of the analysis of vehicles according to their type according to the elements that define the frontal area such as headlights or the light grid, these being much more feasible in processing with the help of video cameras installed at the level of the infrastructure. 

Through these elements, we can later extract characteristics related to homogeneity, and entropy, but also contrast, validating the experimental method through increased accuracy. It can be said that by including the negative events that cause traffic accidents, the collision with other vehicles can be identified through such procedures. The importance of classifying a characteristic region that belongs to the contour of the vehicle can sometimes lead to false detection. By approaching with the help of YOLOv3 and going through LS in multi-layer with filtering of anchor points according to the centroid of the image, the accuracy in the external environment is much higher. The experience gained in projects that addressed road safety issues and the implementation of systems based on visible light communications, as well as radio frequency, is extremely useful in the approach to the field presented. The fusion that can be created at the infrastructure level by combining the data received from different sensors is reflected by the quantity and complexity of the performed calculations. It can be said that the results presented in the article highlight the importance and necessity of implementing a system based on classifiers, with the mention of the implementation within an intelligent system that provides real-time information to both traffic participants and the road infrastructure, with the aim of reducing congestion, accidents, but also the density of vehicles in urban areas. The structure of the article is organized as follows.

Section 2 aims to debate current approaches, but also briefly presents some reference works for specialized literature in the field of vehicle classification. These classifications are extremely important in terms of utility and applicability within applications dedicated to road infrastructure, traffic optimization, but also management and management systems. It will also include the presentation of sensory solutions implemented at the level of the road infrastructure, but also on the road surface. Section 3 presents the efficiency of using classification solutions through neural networks, simulation models, and characteristics of the training sets. Section 4 refers to the aspects related to the performance of the training sets, the outline of the experimental results, but also comparisons with other approaches. In Section 5, the conclusions and highlights of the article are presented, as well as future directions, and implementation.

## 2. Debates Regarding the Taxonomy of Vehicle Classification and Applied Technological Approaches

It can be said that the subject addressed needs a thorough study and a breakdown of the vehicle classifications and a taxonomy of these systems, the details of each one, the technical description, and their performances are necessary. The classification of these systems is performed in three classes, and they differ according to the area in which they are implemented. Classification of vehicle analysis systems, systems based on sensors, and modules that analyze the infrastructure, and systems that can be installed at the level of the road surface, at the level of curbs or traffic lights. Implementation of systems based on piezoelectric sensors [12], magnetometer [13,14], vibration sensors [15]. Data extraction is performed incrementally, optimizing each set of information received from the sensors, including vehicle size or length, number of axles, and unique characteristics rendered by the received signal or waveform. Some of the systems installed at the level of the road infrastructure present results that highlight the high accuracy of vehicle classification, an aspect supported by the close contact between the area where they are installed and the vehicles in motion, memorizing the signs and the way the vehicles move. One of the most important factors that disadvantage these systems is the high installation and maintenance cost, since the road surface requires stripping in the area where these sensors are installed. Costs increase significantly in terms of disruptions created in traffic due to traffic restrictions, but also many other aspects. Instead, the systems installed at the road infrastructure level address the cost problem through vehicle classification schemes by analyzing the traffic through sensors installed at the edge of the roadway. Some of these systems use magnetometers, accelerometers [16], but also acoustic sensors [17]. 

**Figure 1:**
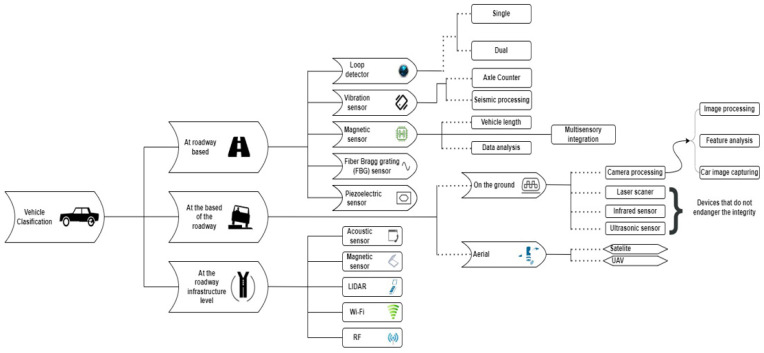
Vehicle classification systems diagram.

The [18] studies used advanced sensors, such as LIDAR (Light Detection and Ranging) and infrared sensors [19,20], but also Wi-Fi receivers and transmitters [21]. In addition to these approaches, the benefits of such a much simpler intervention at low costs cannot be precisely highlighted in terms of performance or analysis accuracy, since, for increased accuracy, the sensors must be installed at certain distances from the road segments and in certain positions so that they facilitate an analysis as accurately as possible. Figure 1 presents the diagram of vehicle classification systems.

Perhaps one of the problems that is difficult to solve with standard application concerns is the accurate classification of overlapping vehicles. The need for a calibration algorithm for the data received from the sensors is imperative, reducing the impact created by the background noise, these are also disturbing elements. The last category of systems that are installed at the upper level of the roads are either satellite or aerial, and their capacity is to cover several lanes on extended surfaces. For example, in the case of unmanned aerial vehicles (UAVs) or satellites they have a greater weight to be used in such applications than the standard variants [22]. The most widespread technology to deal with the highlighted problem is the one based on cameras [23,24]. Although this camera-based approach offers extremely high accuracy in the classification process, its performance decreases in direct proportion to mobility, weather conditions, but also noise and lighting sources. The technological advance in addition to coming with extremely many challenges in terms of automation and digitalization, at the same time new problems arise, one of them concerns the privacy of the driver, maintaining their integrity, and a factor of discretion. In this direction, various sensor packages, infrared [25], laser scanners, and GPS guidance systems [26] have been incorporated into the systems. It can be said that following the presentation of the taxonomy of systems dedicated to vehicle classification, which provides an overview of the field, the following sections will focus on research and development issues, technical challenges, contributions from other research groups, but also design aspects, hardware and software capable of approaching the problem at hand in a different way.

### 2.1. Debating the Current State of In-Roadway-Based Vehicle Classifications

The discussion of the current stages and developments carried out by other research groups in the direction of road-level vehicle classifications highlights certain aspects through which we identify both key points for future studies and also the obstacles encountered by them. Reviewing each system at a theoretical level leads to a set of conclusions regarding the approaches in this article. Although not part of the component developed in this manuscript, loop detectors are used in the classification systems of vehicles traveling on public roads. Thus, the components and results obtained by other researchers who have developed vehicle classification systems using different types of sensors are briefly presented. We address the characteristics between vehicle classification systems and briefly highlight aspects from these manuscripts in Table 1.

**Figure 2:**
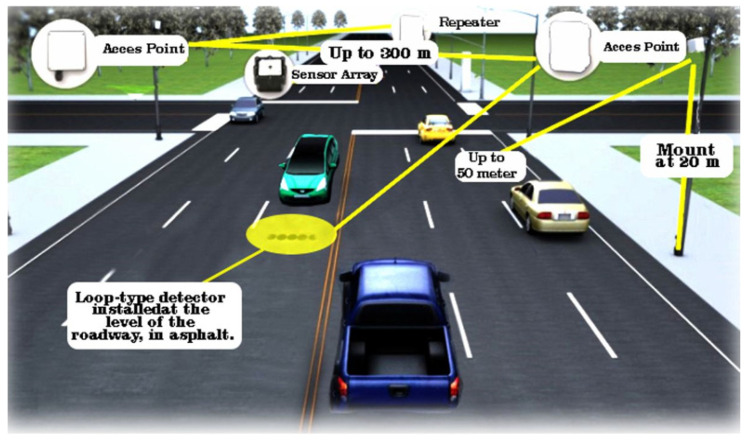
Representative diagram of a loop detector prototype.

In the case of an inductive loop type detector, it is extremely often found in traffic monitoring systems, vehicle detection, traffic flow, but also in vehicle classification [36]. The main component of this type of system consists of a coil of wire embedded in the running surface, see Figure 2. It can be said that it captures the change in inductance, subsequently generating a time-varying signal, only when a vehicle passes over it. Therefore, the characteristics of the signal, such as amplitude, frequency spectrum, or phase, varies according to the classes of vehicles passing by. In other words, these characteristics are unique to the signal and are also known as magnetic profiles [37] for classifying vehicles. Developments so far have led to the implementation of two types of loop detectors, in the first phase depending on their installation method, loops are cut with a special device or perforated loops. In the first one, the cut loops require a special tool that uncovers the running path and embeds the loop wire, and then the stripped space is filled. The second version does not require stripping the running surface and embedding the coils in the asphalt. The wire is mounted through a PVC mold. These loop detectors can also be classified according to the analysis modality, single and dual loop, which reflects the number of detectors used for classification. Dual loop detectors can only measure the speed and length of a vehicle based on the predetermined longitudinal distance between the two dual loop detectors. Even the current developments that are based on automatic learning technologies have produced new challenges through the appearance of devices of this type with automatic learning for the analysis of the magnetic signatures of vehicles that pass over such devices. There are studies [38] in which a backpropagation neural network (BPNN) is adopted for the purpose of classifying vehicles. Thus, based on the observations that low classification accuracy for loop detectors is attributed to data sampling of raw signals containing noises, a Discrete Fourier Transform (DFT)-based algorithm is used to brush and eliminate background noise [39]. 

When analyzing the principal components (PCA) it contains, we highlight that it is used to reduce the size of data that does not contain noise. These PCA features highlight the aspects of the variations in the heights of a vehicle train that traverses the area. Thus, the initial PCA result is entered into BPNN with three classification streets in five classes, identified as follows: motorcycle and bus, minibus/van, pick-up/truck, and vehicle/SUV. These systems, as well as the processing method, reached an average accuracy of over 90% in most studies.

### 2.2. Debating the Current State of Over-Roadway-Based Vehicle Classifications

A vehicle classification system at the roadway level requires the installation of sensors and systems above the roadway, thus providing non-intrusive solutions without the need for stripping and mounting equipment on the road surface. The capacity of these classification systems is to cover many more lanes and, depending on the technology used, even entire road segments. Discussing the current status of camera-based classification systems using aerial technologies and platforms, drones, satellites, and overhead cameras. The advantages of camera-based systems have many advantages, including the accuracy of the classification process, and the extremely large coverage area; the disadvantage is related to privacy issues. The presentation of privacy issues is seen through the use of infrared sensors [40] or laser scanners [41]. The privacy aspect can also be seen in the case of the systems installed by the authorities of each country which monitor the speed on the highways, but also the verification by the registration number of the periodic technical inspection. It can be said that the most used sensor for vehicle classification systems is a camera [42,43]. Thus, a camera provides much more complete information in terms of classification, and aspects, such as visual characteristics, dimensions, or geometric shapes that a car has [44]. In relation to the classic systems installed at the level of road infrastructures, those based on video sensors come with a plus in terms of processing power and the way of classification.

**Figure 3:**
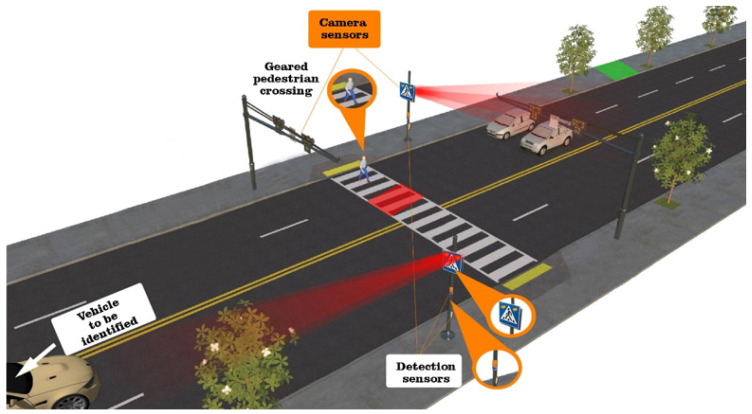
Representative diagram of a traffic monitoring system based on cameras.

Regarding the general operation of a vehicle classification system that is based on the video camera, it has the role of capturing images of passing vehicles, and subsequently, the process of extracting features from those images is carried out with the help of an algorithm in order to achieve the classification. These camera-based systems can, in turn, be classified according to the way in which the video/photo recording is carried out, namely through characteristics related to the exposure time, filtering, and cleaning of the image of external noises. The trend of the last years is related to the improvement of automatic learning techniques applying ways of automatic extraction of features through classification models (Figure 3). Previously developed systems use simple classification models, based on SVM and KNN processing and decision trees, machine learning algorithms, and deep learning are also part of the current approaches. The research group of Chen et al. focused on efficient vehicle image registration [45]. They adopt the Gaussian Mixture Model (GMM) [46] and the shading removal algorithm [47] in order to reduce the negative impact on the classification created by this type of noise, camera vibration, and lighting. The use of Kalman filters is highly prevalent in vehicle tracking and their classification by SVM. The practical experiments were carried out by research groups in the UK and classified about five categories, such as vans, cars, motorcycles, and other unidentified vehicles. The classification accuracy report achieved a percentage of approximately 95% accuracy.

Approaches where images of vehicles were captured but required different interpretations, as they suffered from dynamic changes, having problems caused by lighting changes or headlight reflection in order to improve accuracy. The application of methods by which the chroma of the background is decreased, but also the reduction of the segmentation thresholds, dynamically adjusting the process with the aim of keeping only the gradient differential characteristics in the background. In this case, the approach has two steps to derive the spatial and temporal characteristics of a vehicle. Therefore, 2D estimates of the dimensions of the vehicle are generated, later they take shape and arrive at a 3D exposure in order to obtain a much more accurate classification. The most common vehicles considered for classification are two-wheelers and heavy and light vehicles. Accuracy in the case of such processing and classifications reached 93%. Using pixel bands across periods creates a spatial image. Therefore, vehicle detection and classification by multiple SITs increases the degree of error due to the occlusion created. 

Thus, identifying effective features from an image containing vehicles is a new challenge in camera-based processing and analysis. Classifications made by HOG (Histogram of Oriented Gradient) adjustment methods substantially adjust the results and classification performance. More precisely, KNN is used in the analysis of features based on shapes, features of the elongation type, shapes of any type, and SVM in the characterization of HOG. These methods are used in a combinatorial mix, a summation, and product rules, the first method aims to determine the vehicle class so that the sum of the two creates probabilities for maximized classifiers, and the product is determined by the result of multiplying the two probabilities. Table 2 shows a number of the characteristics of the vehicle classification systems installed above the taxiway.

CNNs are proposed for the design of unsupervised learning mechanisms and for efficient filtering, managing to extract certain features that deform a vehicle. The second stage of the classifier captures the same elements and forms comparison matrices between the two instances. Using Softmax Pro 7 classifiers provides probabilities for each vehicle type. The most important aspects regarding the mix used consisted of the processing of over 10,000 images of vehicles with approximately six types, including trucks, sedans, minivans, minibuses, and SUVs.

## 3. The Efficiency and Versatility of Classification Solutions through Neural Networks

**Figure 4:**
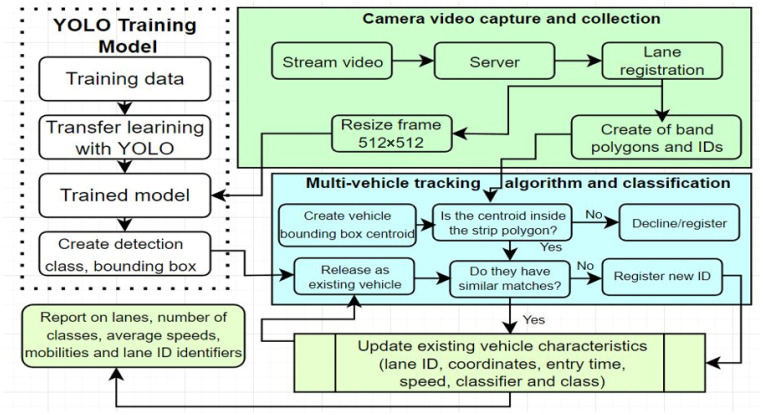
Diagram of vehicle analysis and classification model.

According to the analysis of the specialized literature, several studies highlight that classification and safety surrogate models have been performed because they have achieved data extraction capabilities even from microscopic traffic models, such as Paramics or VISSIM (Verkehr In Städten—SIMulations Modell). Traffic simulation models are solid foundations in the field of engineering and traffic safety compared to existing exposures, notable results are seen in dedicated field projection in the extraction of vehicle trajectories. Thus, the need to introduce an algorithm based on fine-tuned neural networks in order to detect and subsequently classify vehicles in real-time can reach 6–7 identification classes (bus, bicycle, pickup truck, trailer truck, taxi, and car) being only the first part of the characteristics of such an algorithm. Its ability to measure individual speed, calculate the average speed per time interval, track the movement of each vehicle, and also count them [57]. To summarize the general method, video streams are received from road infrastructure surveillance cameras using 4G/5G broadband technologies and others. The video capture is streamed to a computing unit with processing power using a streaming protocol, delineating road lane boundary polygons. After the camera streams are stabilized and the road lanes are registered, the training model detects, classifies, and, at the same time, tracks the vehicles that enter the bounded area in the form of a lane polygon. This algorithm can be identified as a multi-vehicle one in order to track the number, classification, and speed of each vehicle in each lane. The general structure is shown in Figure 4.

### 3.1. Presentation of the Training Dataset

Entrainment of the neural network was performed on two levels in order to minimize the effects of changing the scope. As such, two training sets identified by SR1 and SR2 were prepared. In the case of SR1, it has a larger number of training samples that have been collected from the video stream to train a base model, N1, which has a larger number of steps. Thus, SR2 is based on a much smaller number of samples collected from the outer cameras to create the fine-tuned N2 training model but is based on the redundant data transferred from the N1 level. The two-level training significantly reduces the training time and the effects that domain applicability can have, creating a compromise model, optimized on the principle of classification accuracy and detection speed [58]. On another note, SR1 benefits from information regarding the analysis of roads from the Department of Public Safety Suceava (stream accessible for free from outside), reaching approximately 10 TB of video material, from around 12 cameras within 7 days in the interval of 1 June 2022–7 June 2022. The selection of video material was divided into several training sets that also included samples of the selected vehicles at different positions and times of the day. The video material was converted into image frames with annotation divisions, using a program for manual annotation. About seven classes of vehicles were prepared, including buses, bicycles, taxis, trucks with trailers, and vehicles. The total number of samples prepared for the SR1 datasets is shown in Table 3 in the SR1 samples column.

**Figure 5:**
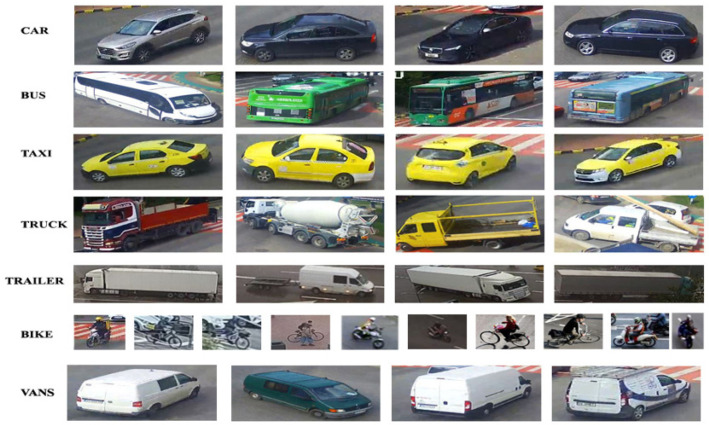
Sampling for vehicles, buses, taxis, bicycles, trucks, vans, auto-trucks.

When we have a much smaller set of labels, as in the case of SR1 being data from cameras used at the road infrastructure level, it is necessary to annotate the images for each of the seven classes. Even if about 7% of the training data in SR1 were not used, they may fall within the recalculation margin, being used for other instances in M1. This testing process increased the number of training samples and the reuse of unused samples. It can be said that the ratios between the test data and their validation have been kept constant, some of the samples of the classes created for vehicle analysis are shown in Figure 5.

### 3.2. Debate the YOLO Algorithm for Detection and Classification

**Figure 6:**
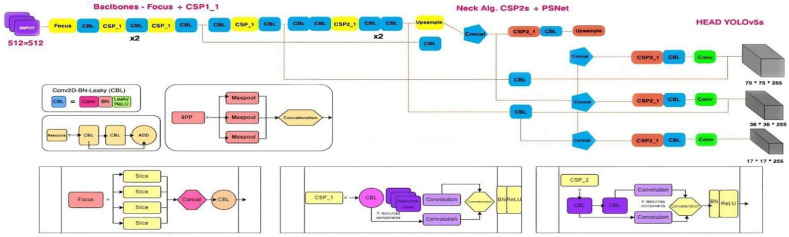
The architecture diagram dedicated to the YOLOv3 network with rigorous adaptations based on the DarkNet-53 backbone.

In this section, the object detection models used in this manuscript and aspects related to the degree of innovation and novelty are discussed. Thus, in addition to the standard method of the YOLOv3 algorithm, a newly derived component has been outlined, entitled YOLOv5s, which has the ability to vary from the smallest dimensions of the learning model. This, in some cases, offers compromises, which end up sacrificing speed and accuracy in the detection process [59]. An obvious difference between these versions concerns the scaling multipliers for both network depth and network width. Through the prism of performance aspects and experience, YOLOv3, YOLOv3-tiny, YOLOv5-large or small were analyzed, all with a comparative purpose. When discussing YOLOv3 (architecture shown in Figure 6), the central basis is a DarkNet-54 backbone with normalization, but also ReLU activation, being used without full connection layers in feature extraction and training of handles from an image. It can be said that a 13 × 13 grid is generated by being projected onto the feature map, and the object is highlighted with approximately three bounding boxes and anchor scales to finally be merged. The first aspect dealing with the final response can be interpreted as when a box has the highest intersection over union (IoU). When we have objects of different sizes, whether small or large, surface features are used, but also depth-based ones that allow detection even if the scales are changed, all based on a residual structure with a reliable connection between all previous YOLO versions. The learning ability of residuals aims to simplify the degree of complexity of the training process and improve detection [60]. The first difference between YOLOv3 and v3t is that the former uses about three scales and v3t relies on only two scales to obtain object prediction. Even in the case of the original v3 version, the ability to manually calculate the size of the anchor boxes was optimized as much as possible, and an approach in the v5s version as well. The approach allows full automation in relation to the initial stage, without the need for the separate calculation of the anchor box. Thus, the YOLOv5s network has, in its composition, several elements of the type of the human skeleton, a vertebral column on which the limbs and the central control area are found. It can be said that for the spine, a CNN is used with the aim of extracting features from the analyzed images even if they have extremely different granularities. The other components are composed of arrays of elements that combine feature analysis functions and delineate anchor boxes from prediction classes. Therefore, the major advance that YOLOv5 has is that it can integrate anchor box selection processes by feeding them into the network, through machine learning capabilities with the best anchor boxes relative to the training dataset [61].

In the case of the v4s core, a CSP-type network was used to aggregate PANnet-type paths with spatial pyramidal blocks, the core neck has the ability to generate characteristic pyramids that help the overall network and can analyze objects at different scales and sizes. Thus, using pyramids and SPP or PANnet block features can substantially increase the identification process even on unseen data. A key integral component of the analysis and classification process is anchor box selection within the neural network, as it has the ability to automatically learn the best anchor boxes for specific training sets [62]. Contact structures were added between the elements that analyze the depths from the common characteristics between YOLOv5s and v5l, which ended up converting the image to a smaller number of depths, sacrificing, in some places, the accuracy, but increasing the speed. When evaluating YOLO networks, an improved intersection (*IoU*), specifically generalized join-intersection (*GioU*) was used for the loss function:

(1) $$L_{GIoU}\left(q\right)=1-IoU+\frac{\left|F\left(C\cup B\right)\right|}{\left|F\right|}$$

where *C* and *B* are bounding boxes of the true values, respective of the prediction, F represents the smallest circumscribed rectangle between *C* and *B*, and *IoU* represents the intersection between *C* and *B*. Thus, the major improvement of *GIoU* compared to standard *IoU* is that it can define a minimal closed area *F*, so the boundaries for *C* and *B* are included in *F*. When we talk about *GIoU*, it then calculates the area of *C* and *B* as not being included in *F*, although this would be proportional to the entire area of *F*.

The next stage after the training process of the YOLO models concerns the collection or recording of the streams received from the cameras installed at the level of the road infrastructure with the help of which the tracking models of the individual classes of vehicles were trained. The developed vehicle tracking and classification algorithm create a centroid tracking gradient that takes a predicted class and generates a bounding box for a predefined pattern, performing the task of computing each road polygon into equal segments. It can be said that the method can highlight the superior performances in order to calculate the degree of matching. Each vehicle uses a trained model and benefits from a vehicle class, after which a vehicle bounding box is obtained for a video recording. These bounding boxes and lane polygons with unique IDs are captured within the algorithm to recursively process multiple vehicles. Part of the processes that the algorithm follows are based on a series of rules. The first rule is the one in which the centroids of the bounding boxes are calculated, and later the degree of matching in the lane polygons is checked, in the case of non-conformity the data are the response and definitely, the vehicle class no longer reaches a process delimitation. In other words, if the verified vehicle matches an existing vehicle in the training dataset it is registered with a new ID and annotated as compatible with the existing feature granularity. After updating the features for an ID, it analyzes the lanes and makes the vehicle part of that lane’s polygon.

## 4. Results and Discussion

In this section, elements related to the performance of the trained networks, the methods, but also the accuracy obtained in the analysis and classification process are presented. Thus, the accuracy of the training and validation data for the YOLO models are compared with the first training instances that have an extremum fine tuning to highlight the degree of domain drift. Some of the experimental results of the lower test levels for the training sets are presented in this section in addition to coupling the models created in YOLO with fine-tuning by analyzing data received from cameras installed at the infrastructure level. To make a comparison, we use precision (P), recall (R) metrics, as well as analysis bases for the curves, this being calculated using the confidence threshold of the model. In the case of a recall, the proportion of all positive data with which the weighted confidence threshold of over 60% is identified identifies it. In the case of precision and recall metrics, they are calculated according to Equations (2) and (3):

(2) $$\mathrm{Precision}\left(P\right)=\frac{TM}{TM+VP}$$

(3) $$\mathrm{Recall}\left(R\right)=\frac{TM}{TM+VN}$$

where *TM*, *VP*, and *VN* represent the number of positive data with a valid truth, false positives, but also those with negative factors are, respectively, false. In the case of the PR curve indicator, it represents the degree of precision for the recall level for the interpolation of the maximum precision calculated in the case of each withdrawal models, there is a grid for the recall coefficient. In the case of mAP, this describes a map of average precision with the number of total existing classes, and, in our case, they are seven in number. In conclusion, AP summarizes PR curves that are defined with average precision over sets of ten recall cycles at approximately equal distances.

(4) $$\mathrm{AP}=\frac{1}{11}\;\overset{1}{\underset{r=0}{\sum }}Pmax\left(r\right),\;r\in \left[0,0,0.1,\ldots ,1\right]$$

(5) $$\mathrm{mAP}=\frac{\sum AP}{n}$$

**Figure 7:**
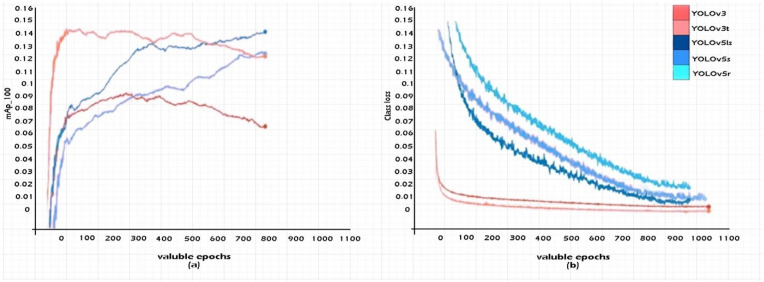
Simulation training results of YOLOv3, YOLOv3t, YOLOv5ls, YOLOv5s, and YOLOv5r on the SRI dataset. (**a**) mAPT at *IoU* = 60; (**b**) classification loss.

The description of the training sets for the YOLO models from the SR1 data series and the results obtained are presented and detailed in this section, the data are packaged in a separate set. Pre-training difficulties have no contact in the initialization of training sets. Applying a random increment to the data for the training period, led to a variable scalar inclusion of over 0.5–1 with a shift of 0.1 for a horizontal angle of approximately 180°. Through the valorization process of the ratio of hue and saturation, it was completely modified segmentally with filling factors of up to 0.5. The input images were resized to 512 × 512 pixels before being transmitted over the network. In the case of the learning set, a threshold of 0.03 was set; for the final learning rate, a threshold of 0.1 was reached, and the impulse for weight reduction through granularity was reached at a filling factor of 0.005. The scaling of the stochastic gradient played the role of the model optimizer, these being classified into batches for each set of approximately 800 frames. The hardware requirement for the simulations were identified as a unit with 256 GB Ram and two I9 processors with two NVIDIA 3060 GPUs (Nvidia, CA, USA). In the case of all five networks created at the simulation level, the dimensions of the anchors and their derivation were characterized by deriving the set of training using k-means. The standard training level on the SR1 set trained 1500 data on gradient maps with high factors. The generation of an IOU map with a loss of class and the training data with the obtained accuracies are exemplified in Figure 7. In the experiments, the chosen and improved algorithm outperformed the other implemented standards. The latest generation models showed an exponential increase, while the others showed a weighted decrease.

In order to obtain an additional comparison between AP and mAP, the five YOLO networks and their derivatives were tested based on the trained dataset SR1. In Table 4 are presented the AP distributions of the prediction against the validation datasets that were provided within the input dataset SR1 for each class of vehicles: cars, buses, taxis, bicycles, trucks, pickups, trailers. A significant difference can be observed in the case of networks formed and outlined based on YOLOv5s and v4r (with double loop recall function).

**Figure 8:**
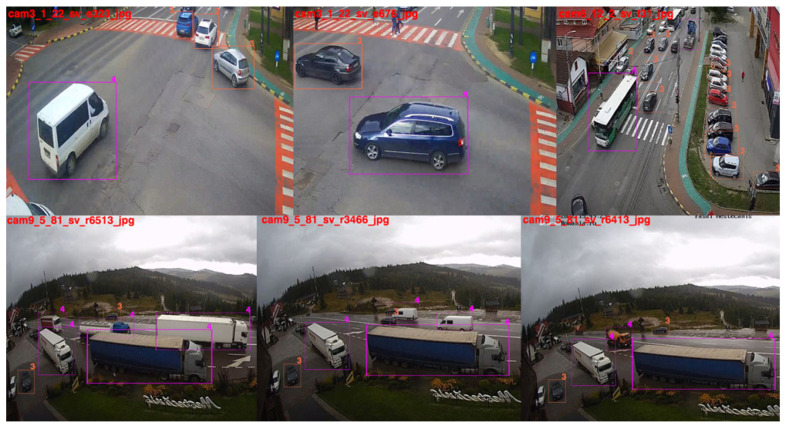
Analysis of the results obtained for the YOLOv5r training model using the SR1 test dataset. Analysis of established classes for vehicles, buses, taxis, bicycles, trucks, vans, auto-trucks.

The results extracted from the samples provided by running with the YOLOv5r training model on the TR1 test set are presented in Figure 8. We note that these test samples in the image are slightly different from the ROI polygons, they extract the tracking gradients generated by the created method. Therefore, ROI placed at appropriate distances can gradually reduce false detection, the result being directly proportional to the exposure created by the detection in the section. There is an alternation, by which the movement in the field changes the accuracy of the training models, which has extractions in the explored section. The exposure for these extractions was carried out in the same environment in which the initial analysis models were analyzed and created, i.e., conditions of intense traffic, varied brightness, and unstable meteorological conditions. The flow of vehicles transiting the analyzed areas exceeds 500 units per minute every 3 min for each direction of travel.

### 4.1. Testing and Analyzing the Created Models and Training Sets for SR1 and SR2

According to the obtained results, it was shown that the training networks performed extremely well for the SR1 dataset for a mAP_60, with 77–82% accuracy, some aspects, such as volatility and weather conditions, caused that, in some cases, the collected image frames do not contain an expected granularity. Therefore, testing the networks in a synchronous mode, both SR1 and SR2 were able to highlight that the sampling is gradual and the classes produced for each set of analyses do not show anomalies for the APs of the incident classes. Thus, Table 5 shows the performance and accuracy characteristics for subdomain switching in fine-tuned classes.

In order to minimize the effects of the domain dynamics approach, the five networks were adjusted to be able to process the training data from the SR1 and SR2 sets using transfer learning as well. It can be said that these fine adjustments are part of the second stage of preparation, different epochs that sum up over 1000 entities as the first level of preparation. When we introduce the process of fine-tuning with high gradation on raw datasets, we can obtain up to seven times the hAp factor for each model, as shown in Table 6, where YOLO performances are. Another aspect addressed in vehicle classification analysis is the classification factor and its loss when applied to training sets. In other words, the loss, if it tends to reach below the 0.07 threshold, may blur some of the defining features and subsequently led to poor performance or misclassification. It can be said that a reason for such an anomaly is the mAp graphic index through which the losses converge towards a gradual fading inside the formed centroids. Thus, standard YOLO models are much more stable in the case of performance for small datasets and epochs that do not exceed 300 units, subsequently decreasing depending on the version involved in the detection process. In such conditions, the adjustment of the algorithm, but also the involvement at the architecture level, highlights a smaller dimension in processing, an extremely important aspect in the tracking and classification of vehicles.

It can be said that in the case of fine adjustments, substantial and sometimes significant improvements in accuracy can be observed, although there are very many approaches that can further increase the degree of accuracy. These highlighted values reflect the appearance that they are the result of calculations based on full-frame detection of the image. There is the possibility of anchoring the centroid only on areas with a distinct gradient and applying HOG to those areas, an aspect that significantly reduces processing time and accuracy, but decreases performance in some cases when the analyzed image does not benefit from all the necessary characteristics, such as brightness, FOV angle, conditions such as unfavorable weather conditions. In a word, if we limit the detection area and avoid objects from a certain distance from the analyzed area, it can considerably increase the accuracy, because certain objects, if the human eye does not visualize them, means that they have a large enough distance to favor our detection. It can be said that in the case of fine adjustments, substantial and sometimes significant improvements in accuracy can be observed, say that instead of segmenting the video stream we can apply techniques for image processing with user-defined thresholds, creating polygons and gradation bands that act with the detection process only in those areas. These polygons can exponentially increase the detection and decrease the false detection factor, subsequently facilitating the calculation of speed or the generation of reports of restricted lanes.

### 4.2. Comparative Analysis of the Proposed Approach in Relation to the Simulation of Urban Mobility

**Figure 9:**
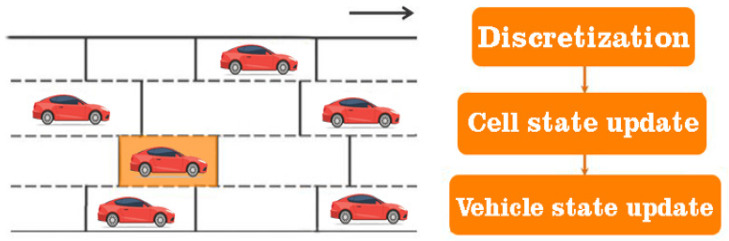
Illustration regarding the macroscopic analysis standard in relation to traffic on separate lanes and cells. Identification of each state in the updated cell through parameterized solutions.

Even though there are very many types of vehicle analysis, even prototyping that is based on macroscopic or microscopic models where they expose the traffic to extremely exponential densities in relation to the potential of the infrastructure, cannot rationalize the problem under discussion. The approach proposed in the article is based on an ad hoc created model applied to the existing video infrastructure at the track level. There are premises for hybridity between continuum, and macroscopic methods, these being based on agents, an aspect that excels in the analysis of individual vehicles. Implementation at the infrastructure level even needs an individual approach through intensive analysis of the infrastructure and the capabilities that the runway provides. Therefore, the relationship that can be exposed through the features that the proposal in this article has is one of dynamic and automatic communication, synchronization between modeling methods, and display on levels of detail. It can be said that traffic flows can be treated as crowds within the simulators, only that the flows differ from time T, in relation to individual behaviors, these having similarities that can interact differently in the driving process. Thus, computer graphics were outlined based on existing datasets, and crowds were simulated with a restricted area, but with a dynamic behavioral stage, but with the modeling of a crowd of a dynamic of agents with movements controlled by microscopic procedures, seein in Figure 9. In order to be able to represent part of the previous analyzes in a simulative form, a series of executions were carried out through which some of the varied characteristics were identified as random in the context of vehicle classification dynamics. Each running lane was identified as having approximately 30 min of running time within the network and half of that time was subsequently dedicated to surrogate data collection. Within the simulation, much shorter durations were proposed in relation to the practical evaluation of the traffic through the lens of the imposed temporal constraint, but also of the aspects related to the continuous analysis of the traffic from the outside environment.

In the first behavioral analysis, 8432 pairs of synchronous data with vehicle identifiers were processed, identifying approximately 324 conflicts that are predominant in a statical analysis. Therefore, the iterative assumption in relation to the simulations during peak hours imposes a factor of 0.97 of unusual traffic volume, referring to an average number of 11563 vehicles for the analyzed period.

**Figure 10:**
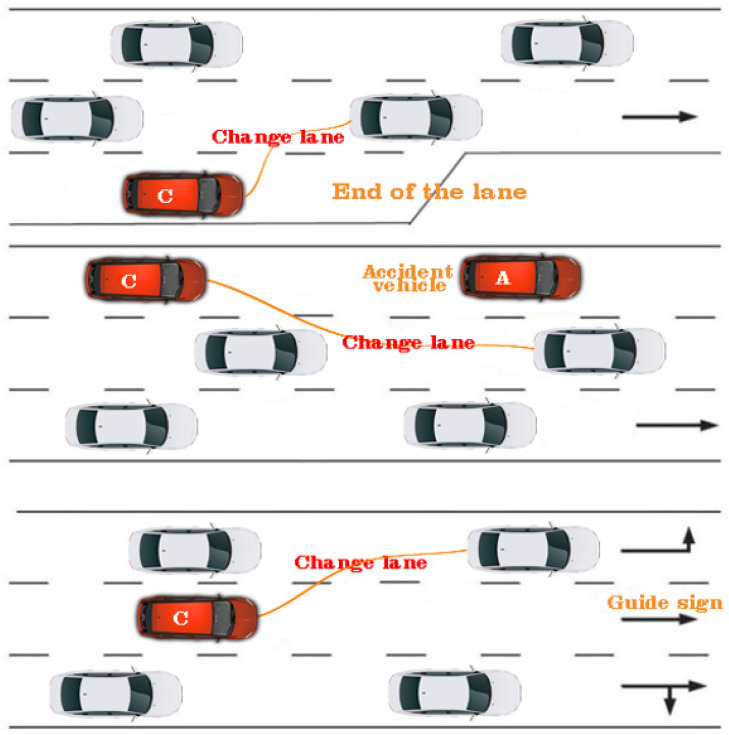
Illustration regarding the analysis and predictability model of urban traffic in conditions of increased density and accident or lane change.

Even from the first series of tests, it can be seen that the simulation time and the processing time can have a similar ratio to the evaluations presented in the manuscript that exceed 120 h through the lens of the two datasets, SR1 and SR2, even if they are in distinct replications. Thus, there are approximately 1244 conflicts out of a total of 42,321 points, even if the length and width characteristics of the vehicles were also loaded. This aspect also involves the direct arrangement of predictive analysis classes with replicated data from SR1 and SR2 in a neural loop to perform the prediction. It can be said that replicated type implementations constitute a certain dimension of static samples, see Figure 10.

Some of the microscopic models can produce uncontrolled movements of the vehicles at an extremely high level, so each vehicle must be treated as a discrete agent capable of satisfying the basic rules of the governing algorithm. In this case, a series of microscopic iterations were developed, which were capable of carrying out simulations specifically for urban traffic, providing increased flexibility for modeling the heterogeneous behaviors of these agents. It can be said that in some simulations where the track is discretized in several cells, the model must determine the next movement of the vehicle from the current cell to the next one. However, due to its discrete nature, the generated virtual traffic cannot fully reproduce the events and behaviors in the traffic; through the prism of these factors, we can say that an exact representation of the proposal described in the manuscript cannot be applied at the simulative level and vice versa. The movement of traffic flows is not the same, because everything depends on the drivers’ intuition and their behavior. We point out that the article proposed definition and analysis by classifying vehicles in congested urban traffic, the model being applied directly to the real-time analysis from the surveillance cameras installed at the level of the infrastructure. The application of the same simulative principle was also carried out in a virtual analysis environment, according to the previous presentations, to highlight the number of vehicles, but also how many of them were classified at the simulative level. To expose this approach most feasibly, different traffic levels were designed with several traffic lanes, but also with access ramps. At the simulative level, the traffic control is programmed, and the production of events is equally predictive through the prism of outlining acceleration and deceleration strategies, programmed turning, and maintaining a behavior. All data it are presented in Table 7.

The interpretation of the results can be performed in only one way, which an exact analysis of the traffic transposed at the simulation level, depending on n factors that can change the state and behavior of the simulations. The interpolated IDM model applied within urban road networks may have a different description in relation to reality. Therefore, the results obtained support the proposed algorithm and the approach represented in the article, but in an ideal situation, without analyzing the traffic and infrastructure for at least 1–2 months to observe if there are repetitive behaviors, similar waiting times, peak hours and congested traffic depending on the time interval and time of day. Even the process of acceleration and deceleration by 1–2 km/h can be an influencing factor in the process of analysis and processing of the traffic model, in terms of the classification of vehicles, the total number of vehicles was also kept in the case of these simulations, and the results are as expected. If we have night traffic, the process is rarer in terms of traffic density, and during the day the weights are close to real cases.

## 5. Conclusions and Future Works

In accordance with the results obtained and the development achieved, we can thus conclude that the future directions regarding approaches are extremely vast through location analysis, but also though road transport automation based on camera and video processing, and artificial intelligence. These elements are extremely important in the development of ITS systems, and, at the same time, are challenging in order to achieve some performance in terms of classifiers and low transit times. Therefore, this article comes as an alternative to the existing detection and classification methods, but brings new elements in terms of the results obtained and their validity. The ability to process very large datasets and visualize training sets with over 10,000 entities highlights the accuracy of vehicle classification that can be considered in larger development. The integration of over 50,000 learning sets was prepared and recorded on the transit routes of Suceava county. The datasets were used to train a highly versatile deep learning network capable of handling extremely large databases. Fine adjustments were made through transfer learning and collaborative neural processing techniques with the aim of being able to implement an ITS-type system in the future that will include the current software component. This training process is divided into two stages that enable leveraging and learning from datasets across applied domains. Thus, the process is able to reduce the number of samples dedicated to the training sets, but also the number of iterations, which can implement new systems, without bringing negative effects or requiring the creation or change of datasets between domains of applicability. In another phase, the model was adjusted to combine multiple processing standards to classify vehicles and calculate distances and body features through fine-tuning and deep learning based on the YOLO algorithm. For each of the five architectures, a new neural network and components were assembled which were capable of reducing the conversion time with respect to the spatio-temporal properties of the vehicles, smoothing the dissipation gradient and strengthening the features related to them, including propagation, but also the reuse of already existing features regardless of size and shape. In the case of the five models, the detection and classification tests were performed using the presented algorithm proposal. The YOLOv5r model achieved an overall accuracy of over 90% in the process of vehicle detection and classification, with variable accuracy depending on meteorological characteristics, image quality, FOV, or noise sources. In the main future directions, this knowledge will be applied for the purpose of validating the analysis of vehicles, regardless of conditions, all through direct communication between vehicles and infrastructure, using VLC, 4 or 5G optical communications, through which the development of roads and pedestrians is developed. We want safety systems, systems for monitoring and streamlining the traffic, and solutions in V2V—V2I vehicle systems, but also between cars using DSRC—5.9 GHz.

## Figures and Tables

**Table 1:** Presentation of systems dedicated to the classification of vehicles at the in-roadway based.

Equipment Type	Authors	Accuracy	Vehicle Classes	Characteristics
Magnetic sensors	Xu et al. [27]	95.46%	Sedans, buses, hatchbacks, and others	It used advanced machine learning techniques in order to obtain a classification focused on imbalance effects
	Li et al. [28]	96.4%	SUVs, buses, vans	It focused on sensor fusion of magnetic waveforms being collected from two magnetic sensors located 80 m apart from each other, but in the same driving lane
	Balid et al. [29]	97%	Single-unit trucks, combination trucks, and others	Made a classification based on types of automatic learning using the length of the vehicle as the key feature
Vibration sensors	Stocker et al. [30]	83%	Light, heavy vehicles	Made a unique feature for seismic signals used as key features for feedforward multilayer perceptron (MLP) artificial neural networks for classification
	Zhao et al. [31]	89.4%	Bus, passenger cars, and big tucks with axle	It used the number of axles and the distance between them as the key characteristic, thus classifying two-axle cars that have similar axle configurations using a multi-parameter classifier
	Jin et al. [32]	92%	Amphibian vehicle and wagon	Based on the complexity of the seismic signal, he used a convolutional neural network (CNN) with a long-scale frequency cepstral coefficient (LFCC) matrix, all identified by the key feature type for complex problems
Loop detectors	Lamas et al. [33]	96%	Vans, trucks, vehicles	The procedure based on the spectral characteristics of inductive signatures using DFT
	Liu et al. [34]	99.4%	Regular cars and long vehicles	Work that was based on the analysis of the length of the vehicle using a detector with a single loop, using the theory of speed estimation through distinct classifications
	Wu et al. [35]	99%	Three length classes with boundaries	Dealt with the problem of acceleration from different points and times 0 of the cars transiting the analyzed area

**Table 2:** Presentation of systems dedicated to the classification of vehicles at the over-roadway based.

Equipment Type	Authors	Accuracy	Vehicle Classes	Characteristics
Camera	Chen et al. [48]	94.6%	Cars, vans, buses, motorcycles, and others	GMM was used to eliminate background noise and SVM for classification
	Karaimer et al. [49]	96.5%	Cars, vans, and motorcycles	A mixture of KNN and SVM was used for shape-based feature analysis, and for general HOG features
	Huttunen et al. [50]	97%	Trucks, vans, and small cars	DNN was used to extract the features
	Zhao et al. [51]	97.9%	Cars, vans, trucks, SUVs, and others	To create a visual attention mechanism, they focus on a single part of the car, considering this approach much more relevant
Aerial platforms	Tan et al. [52]	80.3%	Vans, pickups, sedans, trucks	The use of aerial devices equipped with infrared sensors, and at the level of algorithms Inception Model and AlexNet were used in order to achieve the classification
	Cao et al. [53]	90%	Only detection	It has no applicability in the field of vehicles
	Liu et al. [54]	98.2%	Trucks and cars	Using HOG features together with a neural network, this one having only one hidden layer
Laser scanner	Sandhawalia et al. [55]	82.6%	Trucks, motorcycles, trucks with one or two trailers, vehicles with passengers, and others	Analysis and representation of profiles by laser scanning in image form
	Chidlovskii et al. [56]	86.9%	Trucks, motorcycles, trucks with one or two trailers, vehicles with passengers, and others	Characteristics specific to the application area, analysis of the shape of the vehicles, extracted with the help of a laser scanner for classification

**Table 3:** Sampling for cars, buses, taxis, trucks, trailers, bicycles, vans.

Vehicle Type	SR1 Samples	Tag’s SR2	SR2 Samples
**Car**	12,562	3284	2754
**Bus**	5493	133	376
**Taxi**	2341	332	434
**Truck**	1542	426	764
**Trailer**	1158	98	257
**Bike**	2849	678	743
**Vans**	7062	2114	1774
**Total**	33,007	7065	14,167

**Table 4:** Performance of YOLO networks for SR1 training sets and their evaluation and validation.

Classes	Average Precision (AP)				
	YOLOv3	YOLOv3t	YOLOv5ls	YOLOv5s	YOLOv5r
**Car**	0.688	0.832	0.644	0.794	0.888
**Bus**	0.854	0.974	0.933	0.966	0.913
**Taxi**	0.943	0.799	0.875	0.889	0.869
**Truck**	0.870	0.649	0.789	0.856	0.842
**Trailer**	0.772	0.755	0.867	0.744	0.943
**Bike**	0.893	0.876	0.830	0.890	0.790
**Vans**	0.746	0.670	0.776	0.799	0.828
**mAP_60**	0.812	0.843	0.809	0.791	0.890
**Class Loss**	0.003	0.005	0.022	0.031	0.039

**Table 5:** Performance characteristics of YOLO networks on SR1 training sets and evaluating data from fine-tuned SR2 test sets.

Classes	Average Precision (AP)				
	YOLOv3	YOLOv3t	YOLOv5ls	YOLOv5s	YOLOv5r
**Car**	0.481	0.341	0.338	0.336	0.388
**Bus**	0.28	0.422	0.241	0.142	0.13
**Taxi**	0.644	0.364	0.301	0.464	0.569
**Truck**	0.371	0.257	0.238	0.483	0.342
**Trailer**	0.272	0.246	0.308	0.351	0.243
**Bike**	0.493	0.271	0.245	0.146	0.090
**Vans**	0.346	0.152	0.103	0.058	0.028
**mAP_60**	0.282	0.267	0.209	0.291	0.290

**Table 6:** Performance characteristics of YOLO networks training on previous SR1 sets, applied fine-tuning on SR2 with data evaluation and validation on SR2 set.

Classes	Average Precision (AP)				
	YOLOv3	YOLOv3t	YOLOv5ls	YOLOv5s	YOLOv5r
**Car**	0.793	0.715	0.733	0.839	0.791
**Bus**	0.871	0.767	0.657	0.686	0.774
**Taxi**	0.645	0.658	0.765	0.757	0.677
**Truck**	0.741	0.771	0.858	0.674	0.755
**Trailer**	0.845	0.681	0.667	0.858	0.668
**Bike**	0.741	0.759	0.785	0.788	0.436
**Vans**	0.421	0.851	0.691	0.663	0.615
**mAP_60**	0.771	0.634	0.656	0.641	0.610
**Class Loss**	0.010	0.021	0.029	0.033	0.036
**Model Size (MB)**	283.44	35.21	87.36	44.61	20.14

**Table 7:** Traffic analysis and classification of vehicles depending on the time of day, the hypothesis treated at a simulative level.

Classes	Average Precision (AP)			
	Morning Traffic	Midday Traffic	Evening Traffic	Traffic at Night
**Car**	0.361	0.525	0.312	0.419
**Bus**	0.384	0.476	0.231	0.391
**Taxi**	0.318	0.389	0.448	0.489
**Truck**	0.293	0.276	0.374	0.588
**Trailer**	0.351	0.233	0.465	0.549
**Bike**	0.434	0.358	0.388	0.156
**Vans**	0.466	0.451	0.421	0.351
**Class Loss**	0.341	0.239	0.221	0.167

## Data Availability

Not applicable.

## References

[1] U.S. Department of Transportation Research and Innovative Technology Administration (2010). Frequency of Target Crashes for IntelliDrive Safety Systems.

[2] World Health Organization WHO The 10 Leading Causes of Death in the World, 2017 and 2018. Fact Sheet. https://www.who.int/news-room/fact-sheets/detail/the-top-10-causes-of-death.

[3] Ashraf I., Hur S., Shafiq M., Park Y. (2019). Catastrophic factors involved in road accidents: Underlying causes and descriptive analysis. PLoS ONE.

[4] Chang F.R., Huang H.L., Schwebel D.C., Chan A.H.S., Hu G.Q. (2020). Global road traffic injury statistics: Challenges, mechanisms and solutions. Chin. J. Traumatol..

[5] Lee H., Coifman B. (2015). Using LiDAR to validate the performance of vehicle classification stations. J. Intell. Transp. Syst..

[6] Tyburski R. (1988). A review of road sensor technology for monitoring vehicle traffic. Inst. Transp. Eng. J..

[7] Yannis G., Papadimitriou E., Folla K. (2014). Effect of GDP changes on road traffic fatalities. Saf. Sci..

[8] (2021). Mobility and Transport—Curent Trends and Issues Transport in the European Union—Edition 2020 from European Commission Transportation. https://www.consilium.europa.eu/en/council-eu/configurations/tte/.

[9] Corral P., Rodríguez-Mas F., Alonso J.L., Ferrer J.C., Fernández de Ávila S. (2020). A Low-Cost IEEE 802.15.7 Communication System Based on Organic Photodetection for Device-to-Device Connections. Sensors.

[10] He Y., Liu Z., Zhou X., Zhong B. Analysis of Urban Traffic Accidents Features and Correlation with Traffic Congestion inLarge-Scale Construction District. Proceedings of the 2017 International Conference on Smart Grid and Electrical Automation (ICSGEA).

[11] Yousaf K., Iftikhar A., Javed A. (2012). Comparative analysis of automatic vehicle classification techniques: A survey. Int. J. Image Graph. Signal Process..

[12] Rajab S.A., Mayeli A., Refai H.H. Vehicle classification and accurate speed calculation using multi-element piezoelectric sensor. Proceedings of the 2014 IEEE Intelligent Vehicles Symposium Proceedings.

[13] Bottero M., Chiara B.D., Deflorio F.P. (2013). Wireless sensor networks for traffic monitoring in a logistic centre. Transp. Res. C Emerg. Technol..

[14] Li H., Dong H., Jia L., Ren M. (2014). Vehicle classification with single multi-functional magnetic sensor and optimal MNS-based CART. Measurement.

[15] Bajwa R. (2013). Wireless Weigh-In-Motion: Using Road Vibrations to Estimate Truck Weights.

[16] Shen L. (2013). Research on E-Commerce Website Design Based on User Experience—Taking Online Digital Printing as an Example. Master’s Thesis.

[17] Ntalampiras S. (2018). Moving vehicle classification using wireless acoustic sensor networks. IEEE Trans. Emerg. Top. Comput. Intell..

[18] Lee H., Coifman B. (2012). Side-fire LiDAR-based vehicle classification. Transp. Res. Rec..

[19] Odat E., Shamma J.S., Claudel C. (2018). Vehicle classification and speed estimation using combined passive infrared/ultrasonic sensors. IEEE Trans. Intell. Transp. Syst..

[20] Won M., Zhang S., Son S.H. WiTraffic: Low-cost and non-intrusive traffic monitoring system using WiFi. Proceedings of the 26th International Conference on Computer Communication and Networks, ICCCN 2017.

[21] Yang B., Lei Y. (2015). Vehicle detection and classification for low-speed congested traffic with anisotropic magnetoresistive sensor. IEEE Sens. J..

[22] Puri A. (2005). A Survey of Unmanned Aerial Vehicles (UAV) for Traffic Surveillance.

[23] Adu-Gyamfi Y.O., Asare S.K., Sharma A., Titus T. (2017). Automated vehicle recognition with deep convolutional neural networks. Transp. Res. Rec..

[24] Chang J., Wang L., Meng G., Xiang S., Pan C. (2018). Vision-based occlusion handling and vehicle classification for traffic surveillance systems. IEEE Intell. Transp. Syst. Mag..

[25] Noyce D.A., Gajendran A., Dharmaraju R. (2006). Development of Bicycle and Pedestrian Detection and Classification Algorithm for Active-Infrared Overhead Vehicle Imaging Sensors. Transp. Res. Rec..

[26] Cheung S.Y., Coleri S., Dundar B., Ganesh S., Tan C.-W., Varaiya P. (2005). Traffic Measurement and Vehicle Classification with Single Magnetic Sensor. Transp. Res. Rec..

[27] Xu C., Wang Y., Bao X., Li F. (2018). Vehicle classification using an imbalanced dataset based on a single magnetic sensor. Sensors.

[28] Li F., Lv Z. (2017). Reliable vehicle type recognition based on information fusion in multiple sensor networks. Comput. Netw..

[29] Balid W., Tafish H., Refai H.H. (2018). Intelligent vehicle counting and classification sensor for real-time traffic surveillance. IEEE Trans. Intell. Transp. Syst..

[30] Stocker M., Rönkkö M., Kolehmainen M. (2014). Situational knowledge representation for traffic observed by a pavement vibration sensor network. IEEE Trans. Intell. Transp. Syst..

[31] Zhao H., Wu D., Zeng M., Zhong S. (2018). A vibration-based vehicle classification system using distributed optical sensing technology. Transp. Res. Rec..

[32] Jin G., Ye B., Wu Y., Qu F. (2019). Vehicle classification based on seismic signatures using convolutional neural network. IEEE Geosci. Remote Sens. Lett..

[33] Lamas-Seco J., Castro P., Dapena A., Vazquez-Araujo F. (2015). Vehicle classification using the discrete Fourier transform with traffic inductive sensors. Sensors.

[34] Liu H.X., Sun J. (2014). Length-based vehicle classification using eventbased loop detector data. Transp. Res. C Emerg. Technol..

[35] Wu L., Coifman B. (2014). Vehicle length measurement and length-based vehicle classification in congested freeway traffic. Transp. Res. Rec..

[36] Wu L., Coifman B. (2014). Improved vehicle classification from dual-loop detectors in congested traffic. Transp. Res. C Emerg. Technol..

[37] Hernandez S.V., Tok A., Ritchie S.G. (2016). Integration of weigh-inmotion (WIM) and inductive signature data for truck body classification. Transp. Res. C Emerg. Technol..

[38] Meta S., Cinsdikici M.G. (2010). Vehicle-classification algorithm based on component analysis for single-loop inductive detector. IEEE Trans. Veh. Technol..

[39] Shokravi H., Shokravi H., Bakhary N., Heidarrezaei M., Rahimian Koloor S.S., Petrů M. (2020). A Review on Vehicle Classification and Potential Use of Smart Vehicle-Assisted Techniques. Sensors.

[40] da Costa Filho A.C., de Brito Filho J.P., de Araujo R.E., Benevides C.A. Infrared-based system for vehicle classification. Proceedings of the 2009 SBMO/IEEE MTT-S International Microwave and Optoelectronics Conference (IMOC).

[41] Zadobrischi E., Cosovanu L.-M., Dimian M. (2020). Traffic Flow Density Model and Dynamic Traffic Congestion Model Simulation Based on Practice Case with Vehicle Network and System Traffic Intelligent Communication. Symmetry.

[42] Nam Y., Nam Y.C. (2018). Vehicle classification based on images from visible light and thermal cameras. J. Image Video Proc..

[43] Sarikan S.S., Ozbayoglu A.M., Zilci O. (2017). Automated Vehicle Classification with Image Processing and Computational Intelligence. Procedia Comput. Sci..

[44] Asaidi H., Aarab A., Bellouki M. (2014). Shadow elimination and vehicles classification approaches in traffic video surveillance context. J. Vis. Lang. Comput..

[45] Yang M.T., Jhang R.K., Hou J.S. (2013). Traffic flow estimation and vehicle-type classification using vision-based spatial-temporal profile analysis. IET Comput. Vis..

[46] Zivkovic Z., van der Heijden F. (2006). Efficient adaptive density estimation per image pixel for the task of background subtraction. Pattern Recognit. Lett..

[47] Chen Z., Pears N., Freeman M., Austin J. (2009). Background subtraction in video using recursive mixture models, spatio-temporal filtering and shadow removal. International Symposium on Visual Computing.

[48] Chen Z., Ellis T., Velastin S.A. Vehicle detection, tracking and classification in urban traffic. Proceedings of the 15th International IEEE Conference on Intelligent Transportation Systems.

[49] Karaimer H.C., Cinaroglu I., Bastanlar Y. Combining shape-based and gradient-based classifiers for vehicle classification. Proceedings of the 2015 IEEE 18th International Conference on Intelligent Transportation Systems.

[50] Huttunen H., Yancheshmeh F.S., Chen K. Car type recognition with deep neural networks. Proceedings of the IEEE Intelligent Vehicles Symposium (IV).

[51] Zhao D., Chen Y., Lv L. (2017). Deep reinforcement learning with visual attention for vehicle classification. IEEE Trans. Cognit. Dev. Syst..

[52] Tan Y., Xu Y., Das S., Chaudhry A. Vehicle detection and classification in aerial imagery. Proceedings of the 25th IEEE International Conference on Image Processing (ICIP 2018).

[53] Cao X., Wu C., Yan P., Li X. Linear SVM classification using boosting HOG features for vehicle detection in low-altitude airborne videos. Proceedings of the 18th IEEE International. Conference on Image Processing. (ICIP 2011).

[54] Liu K., Mattyus G. (2015). Fast multiclass vehicle detection on aerial images. IEEE Geosci. Remote Sens. Lett..

[55] Sandhawalia H., Rodriguez-Serrano J.A., Poirier H., Csurka G. Vehicle type classification from laser scanner profiles: A benchmark of feature descriptors. Proceedings of the 16th International IEEE Conference on Intelligent Transportation Systems (ITSC 2013).

[56] Chidlovskii B., Csurka G., Rodriguez-Serrano J. Vehicle type classification from laser scans with global alignment kernels. Proceedings of the 17th International IEEE Conference on Intelligent Transportation Systems.

[57] Song H., Liang H., Li H., Dai Z., Yun X. (2019). Vision-based vehicle detection and counting system using deep learning in highway scenes. Eur. Transp. Res. Rev..

[58] Wu C.W., Zhong M.T., Tsao Y., Yang S.W., Chen Y.K., Chien S.Y. Track-clustering error evaluation for track-based multi-camera tracking system employing human re-identification. Proceedings of the IEEE Conference on Computer Vision and Pattern Recognition Workshops.

[59] Bochinski E., Eiselein V., Sikora T. High-speed tracking-by-detection without using image information. Proceedings of the 14th IEEE International Conference on Advanced Video and Signal Based Surveillance (AVSS).

[60] Hou X., Wang Y., Chau L.P. Vehicle tracking using deep sort with low confidence track filtering. Proceedings of the 2019 16th IEEE International Conference on Advanced Video and Signal Based Surveillance (AVSS).

[61] Wende F., Cordes F., Steinke T. On improving the performance of multi-threaded CUDA applications with concurrent kernel execution by kernel reordering. Proceedings of the 2012 Symposium on Application Accelerators in High Performance Computing.

[62] Neupane B., Horanont T., Aryal J. (2021). Deep learning-based semantic segmentation of urban features in satellite images: A review and meta-analysis. Remote Sens..

